# Thermodynamic and Kinetic Modeling Directs Pathway Optimization for Isopropanol Production in a Gas-Fermenting Bacterium

**DOI:** 10.1128/msystems.01274-22

**Published:** 2023-03-27

**Authors:** Jonathan Lo, Chao Wu, Jonathan R. Humphreys, Bin Yang, Zhenxiong Jiang, Xin Wang, PinChing Maness, Nicolas Tsesmetzis, Wei Xiong

**Affiliations:** a Biosciences Center, National Renewable Energy Laboratory, Golden, Colorado, USA; b Department of Microbiology, Miami University, Oxford, Ohio, USA; c Shell International Exploration and Production, Inc., Houston, Texas, USA; University of Massachusetts Medical School

**Keywords:** *Clostridium ljungdahlii*, flux control index, isopropanol, gas fermentation, metabolic robustness analysis, protein cost analysis, thermodynamic analysis

## Abstract

Rational engineering of gas-fermenting bacteria for high yields of bioproducts is vital for a sustainable bioeconomy. It will allow the microbial chassis to renewably valorize natural resources from carbon oxides, hydrogen, and/or lignocellulosic feedstocks more efficiently. To date, rational design of gas-fermenting bacteria such as changing the expression levels of individual enzymes to obtain the desired pathway flux is challenging, because pathway design must follow a verifiable metabolic blueprint indicating where interventions should be executed. Based on recent advances in constraint-based thermodynamic and kinetic models, we identify key enzymes in the gas-fermenting acetogen Clostridium ljungdahlii that correlate with the production of isopropanol. To this extent, we integrated a metabolic model in comparison with proteomics measurements and quantified the uncertainty for a variety of pathway targets needed to improve the bioproduction of isopropanol. Based on *in silico* thermodynamic optimization, minimal protein requirement analysis, and ensemble modeling-based robustness analysis, we identified the top two significant flux control sites, i.e., acetoacetyl-coenzyme A (CoA) transferase (AACT) and acetoacetate decarboxylase (AADC), overexpression of which could lead to increased isopropanol production. Our predictions directed iterative pathway construction, which enabled a 2.8-fold increase in isopropanol production compared to the initial version. The engineered strain was further tested under gas-fermenting mixotrophic conditions, where more than 4 g/L isopropanol was produced when CO, CO_2_, and fructose were provided as the substrates. In a bioreactor environment sparging with CO, CO_2_, and H_2_ only, the strain produced 2.4 g/L isopropanol. Our work highlighted that the gas-fermenting chasses can be fine-tuned for high-yield bioproduction by directed and elaborative pathway engineering.

**IMPORTANCE** Highly efficient bioproduction from gaseous substrates (e.g., hydrogen and carbon oxides) will require systematic optimization of the host microbes. To date, the rational redesign of gas-fermenting bacteria is still in its infancy, due in part to the lack of quantitative and precise metabolic knowledge that can direct strain engineering. Here, we provide a case study by engineering isopropanol production in gas-fermenting Clostridium ljungdahlii. We demonstrate that a modeling approach based on the thermodynamic and kinetic analysis at the pathway level can provide actionable insights into strain engineering for optimal bioproduction. This approach may pave the way for iterative microbe redesign for the conversion of renewable gaseous feedstocks.

## INTRODUCTION

Population growth and changes in energy structure pose a growing pressure on human society. Energizing the world sustainably therefore requires revolutionizing the way we harness natural resources. New renewable technologies include the microbiological upgrading of syngas, a gas mixture consisting of carbon monoxide (CO), hydrogen (H_2_), and carbon dioxide (CO_2_) (1, 2). This technology provides an attractive strategy to valorize low-cost substrates, such as waste streams, into fuels and chemicals in low operating-temperatures/pressures and high product uniformity (3). Coupling this process with cutting-edge CO_2_ capture technologies and photovoltaics-driven electrochemical syngas generation (4, 5) will offer a promising means to produce energy from sunlight, water, and air, without aggravating pressure on the environment (6).

Acetogenic bacteria are being developed as capable chassis organisms for syngas utilization due to their innate Wood-Ljungdahl pathway (WLP), which converts H_2_/CO/CO_2_ to acetyl-coenzyme A (CoA), the primary building block for the biosynthesis of acetate and other fermentation products. The WLP in acetogens can work individually fixing CO_2_, leading to carbon-negative bioconversion, or it can coordinate with the glycolytic pathway for syngas-sugar coutilization with minimal CO_2_ release (7, 8). Thus, highly carbon-efficient biofuel production would be enabled by plugging in a specific foreign pathway that redirects acetyl-CoA flux to the product of interest. Recent development of genetic systems in acetogenic bacteria has made these attempts successful, for example, in the production of isopropanol (IPA), acetone, and 3-hydroxybutyrate (7, 9–12). Nevertheless, most engineering efforts in acetogenic bacteria are in the early stages of development. Some of them rely on laborious trial and error of target pathways, due in part to a lack of comprehensive assessment of pathway properties within the host’s metabolism. The lack of adequate metabolic knowledge hampers pathway optimization a grand challenge. Specifically, artificially engineered pathways in the context of natural metabolism could lack intrinsic optimality. Engineering perturbations in gene expression levels may lead to either fluctuated pathway flux or the disappearance of a stable steady state due to depleted or overaccumulated intermediates, thus leading to a suboptimal metabolic outcome (13). To address this challenge, rational reengineering of gas-fermenting bacteria based on systematic metabolic knowledge will be of potential value.

State-of-the-art rational engineering assisted by constraint-based metabolic modeling has enabled the development of a variety of microbial chasses that host engineered pathways for novel bioproducts (14, 15). Metabolic models typically simulate enzyme-catalyzed metabolic conversions by modeling interactions between genes, enzymes, and reactions. Once metabolic “blueprints” have been profiled computationally, researchers can iterate a design-build-test-learn cycle to identify those genetic modifications that could improve titers, rates, and yields. Although a wide range of computational approaches can be used to correlate gene expressions with fluxes, relatively few approaches seek to describe the pathway properties in all critical dimensions that consider both feasibility, reaction dynamics, and pathway stability comprehensively in an altered environment. It’s also challenging to estimate these models’ predictability for intracellular reactions and cell activities. Earlier computational tools (16) implemented within the COBRA Toolbox (17) were used with the genome-scale metabolic models to enumerate the reactions that should be actively forced through genetic interventions in order to achieve the overproduction of target products. This method can predict the increase, decrease, or elimination of the flux value corresponding to each of the involved reactions. These tools lack quantitative mapping between fluxes and gene expression levels and do not account for kinetic and thermodynamic features governing the metabolic behavior of the host microbe. To address this, new modeling approaches were developed more recently. One of them, known as PathParser (18), combines metabolic models with reaction thermodynamic and kinetic parameters to evaluate the pathway directionality, enzyme requirements, and robustness against expression level changes. Using an ensemble modeling method, this tool can also calculate flux control indexes (FCIs) that describe the sensitivity of metabolism to enzyme expression and can be used to guide strain engineering. However, to what extent PathParser can direct metabolic engineering, specifically in signifying potential modification targets, has not been validated by experimental tests.

In this study, we used a previously developed computational tool (18) to evaluate a redesigned IPA pathway within Clostridium ljungdahlii’s metabolism. The focus of this research is to apply a proof-of-concept application of PathParser to quantify the uncertainties in pathway control that are needed to improve IPA production. We leveraged the proteomics data collected in strain design experiments and tested if our methodology can indeed predict the outcome of strain engineering. The modeling prediction as well as systems biology analysis enabled iterative construction of Clostridium ljungdahlii strains which eventually produced 4.4 and 2.4 g/L IPA under mixotrophic and autotrophic conditions, respectively. It is evidenced that our approach can distill key information in kinetic and thermodynamic parameters and provide executable insights into guiding strain engineering. Given that IPA is among the top blendstocks for turbocharged gasoline engines and has been known to be a drop-in gasoline additive, our work explicitly highlighted the potential and feasibility of rational metabolic redesign and optimization in syngas-fermenting bacteria for biofuel overproduction.

## RESULTS

### Thermodynamics of acetogenic metabolism toward IPA production.

**FIG 1:**
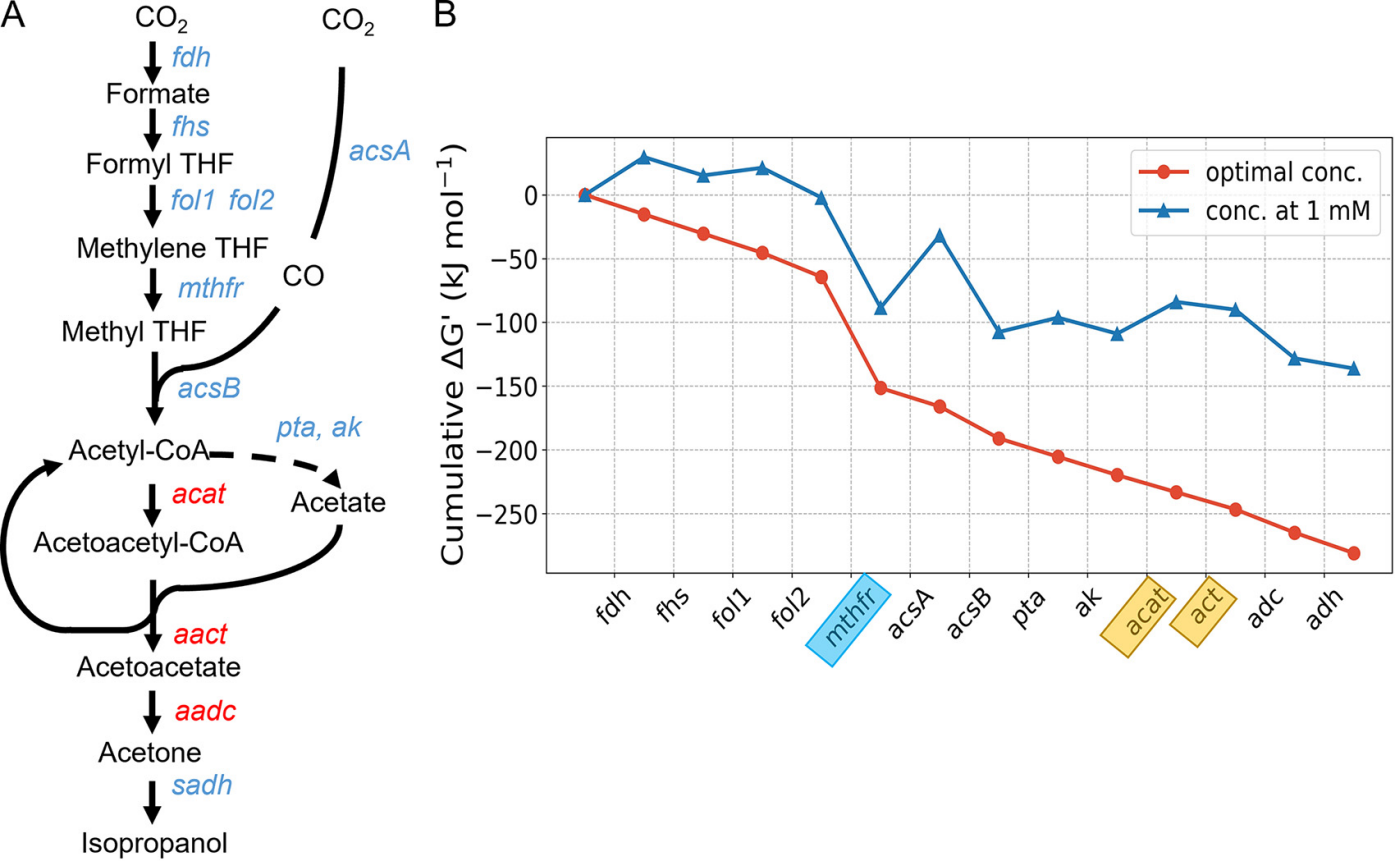
The thermodynamic feasibility of IPA biosynthesis from acetogenic WLP. (A) The WLP and IPA pathway. Heterologous enzymes engineered into *C. ljungdahlii* for IPA production are shown in red. (B) The thermodynamic driving force of the pathway is presented as the cumulative sum of reaction Gibbs energies, ΔG′. The blue line denotes standard Gibbs energies with all metabolite concentrations fixed at 1 mM, and the red line denotes Gibbs energies when the highest reaction ΔG′ is iteratively maximized in the negative direction. The optimized metabolite concentrations are shown in Table S1. The most and least thermodynamically favorable reactions are shown in blue and yellow boxes, respectively.

Microbial IPA production can be derived from the WLP and the central carbon metabolism via acetyl-CoA. This functionality can be achieved by four catalytic reactions, acetyl-CoA acetyltransferase (ACAT), acetoacetyl-CoA transferase (AACT), acetoacetate decarboxylase (AADC), and secondary alcohol dehydrogenase (SADH), acting in sequence (see Fig. 1A) (19). However, the thermodynamic feasibility of the overall pathway within acetogenic metabolism is still poorly understood. We therefore modeled the max-min driving force (MDF) (20, 21) and sought to decipher the pathway potential in a thermodynamic landscape. Specifically, we constructed a thermodynamic model for *de novo* IPA synthesis from syngas with the standard Gibbs energy changes, ΔG′^0^ (or ΔG′^m^ for physiological conditions with 1 mM as the standard concentration) derived from the eQuilibrator database (22). The thermodynamic feasibility of a pathway is impeded by the least favorable reactions. As such, maximizing the minimal minus Gibbs energy change (–ΔG′) of these reactions iteratively will mimic the optimal cellular process toward the overall driving force of the pathway within a physiological range of metabolites (here 1 μM to 10 mM). The optimized driving force of reactions is illustrated as the “downhill” map in Fig. 1. Compared with the unoptimized thermodynamics where metabolites are set with concentrations at 1 mM (Fig. 1B, blue line), the engineered IPA synthesis pathway (Fig. 1B) shows much better thermodynamic feasibility after optimization (–260 kJ/mol versus –150 kJ/mol, both originating from H_2_/CO/CO_2_). The strongest driving force in IPA synthesis is generated by methylenetetrahydrofolate reductase of the WLP (blue box), whereas the weakest driving force lies in the reactions catalyzed by ACAT and AACT (yellow boxes), which need to be engineered for IPA production. It is noteworthy that net pathway flux is fundamentally controlled by metabolite concentrations according to the second law of thermodynamics: ΔG′ = ΔG′^0^ + RT ln Q, where Q is the reaction quotient (i.e., the ratio of products and reactants). During the optimization of overall pathway driving force, we found that the concentrations of acetyl-CoA and acetate reached the preset upper bound of 10 mM (see Table S1 in the supplemental material), which suggests that these two metabolites should be maintained at a high level to guarantee the unobstructed IPA synthesis. Coincidently, acetogenic bacteria as a host meet this requirement well, as they can accumulate acetate, as well as its precursor acetyl-CoA, thus favoring IPA production thermodynamically.

10.1128/msystems.01274-22.1 TABLE S1 Solution of metabolite concentrations obtained in the max-min driving force (MDF) optimization of the Wood-Ljungdahl pathway (WLP) for IPA biosynthesis in *C. ljungdahlii*. Download Table S1, PDF file, 0.03 MB. Copyright © 2023 Lo et al. 2023 Lo et al. https://creativecommons.org/licenses/by/4.0/ This content is distributed under the terms of the Creative Commons Attribution 4.0 International license.

### IPA production enabled by plasmid-based pathway construction and stabilized by genome integration.

**FIG 2:**
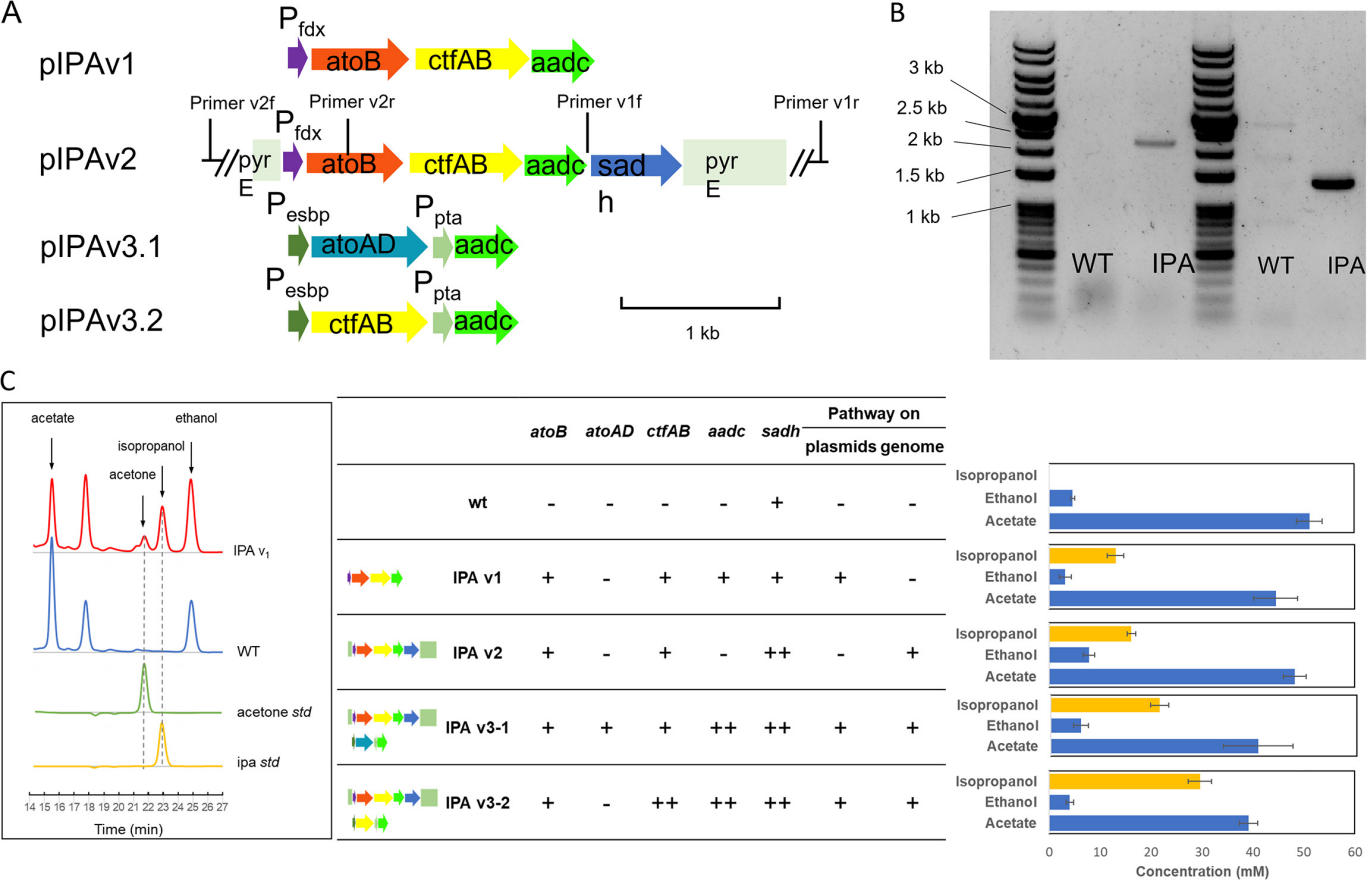
Pathway constructions for IPA biosynthesis. (A) Gene cassettes and expression components on the constructs. (B) PCR results indicating that the IPA pathway on pIPAv2 has been integrated into the genome on the *pyrE* locus. The PCR templates are genomic DNA from the wild-type *C. ljungdahlii* (labeled as WT) and IPA v2 strain (labeled as IPA). PCR products using primer v1f- v1r (left two lanes) and primer v2f-v2r pairs (right two lanes) are shown on the gel picture, respectively. The locations the primers are labeled in panel A. (C) IPA-producing strains and their IPA productivities. Shown on the left are the HPLC profiles of the IPA v1 strain and wild-type broths in comparison to the chemical standards. Dashed lines denote the retention time of IPA and acetone. The presence of IPA pathway genes in various IPA strains and the corresponding IPA titer are shown in the middle and right, respectively. “–” indicates that the gene was not present in the strain; “+” indicates one copy of the gene; “++” indicates two copies in the strain.

*In silico* thermodynamic analysis of the IPA pathway rationalized *in vivo* implementation of pathway construction. To express the pathway, we constructed a plasmid pIPAv1 which contained a codon-optimized *atoB* from Escherichia coli and the *ctfAB* and *aadc* genes from Clostridium acetobutylicum (Fig. 2A) using NEB HiFi assembly. These genes encoding the ACAT, AACT, and AADC, respectively, were placed behind a strong native ferredoxin promoter from *C. ljungdahlii*. The last step, the SADH, can be catalyzed by an endogenous enzyme in *C. ljungdahlii* (23) and thus was not included in the plasmid constructs. HPLC analysis confirmed the presence of both IPA and its precursor acetone (Fig. 2C) in the engineered strain (IPA v1), whereas the parental strain did not demonstrate these two peaks. The initial construct has produced 0.05 g/L acetone and 0.78 g/L IPA in the broth.

A potential risk that could affect the sustainable production of IPA over time was identified. As we passaged the strain, we noticed that IPA levels were getting progressively worse, potentially due to strain degeneracy. A highly expressed nonnative pathway on a plasmid would be genetically less stable than genome expression and might explain the attenuation of IPA productivity over time. We thus redesigned the IPA pathway to be integrated into the genome, targeting the *pyrE* locus. The *pyrE* gene causes sensitivity to 5-fluoroorotic acid (5-FOA) as it converts 5-FOA to a toxic 5-fluorouridine monophosphate (5-FUMP). Disruption of *pyrE* will lead to 5-FOA resistance and thus can be used for selection of genome integration (24). By designing flanking regions targeting *pyrE* and containing the IPA pathway, we successfully transformed these targeted plasmids into the wild-type strain. The transformants showed IPA production, indicating that the pathway carried in the new plasmid is functional (data not shown). 5-FOA selection further allowed us to obtain several 5-FOA-resistant mutants, indicating that the IPA pathway has been integrated into the genome. This was further confirmed by PCR analysis and DNA resequencing using primers that flanked the *pyrE* locus (Fig. 2B). In the second version of IPA strains (IPA v2), 0.97 g/L IPA titer was achieved from YTF medium containing Yeast extract, Tryptone and Fructose, and the IPA production showed fairly good stability over ~10 passages.

### Ensemble modeling inferred metabolic robustness as well as flux control indexes.

**FIG 3:**
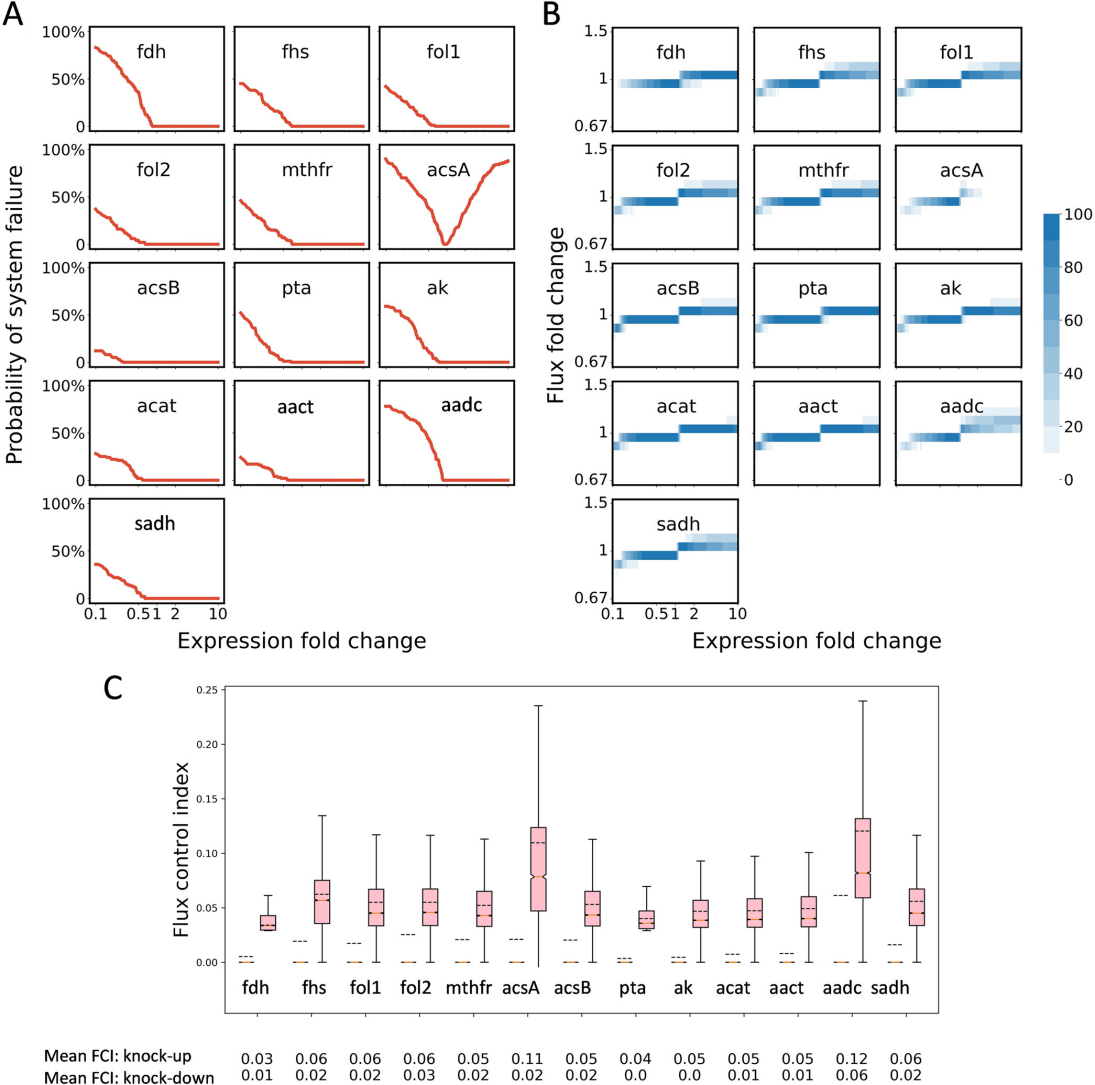
(A to C) Robustness analysis (A) and flux control indexes (B and C) of the IPA biosynthetic pathway. (A) Pathway robustness is represented by the probability of system failure at various fold changes of enzyme expression levels over the reference state. A pathway is considered to be entering system failure when any intermediate is depleted or overaccumulated, and the probability of system failure is calculated as counts in an ensemble of 100 models which were generated with log-uniformly sampled kinetic parameters from the feasible spaces, meanwhile being subjected to the same flux distribution as the reference state. (B) Flux fold changes by enzyme perturbation were evaluated by an ensemble of 100 models which were generated with log-uniformly sampled kinetic parameters from the feasible spaces, meanwhile being subjected to the same flux distribution as reference state. The results are presented as a set of heatmaps, and the color indicates the number of models of corresponding flux fold change at some enzyme expression level for each enzyme. (C0 Based on the visualized results in panel B, flux control indexes (FCIs) were also calculated to quantitatively describe the extent of pathway flux change caused by enzyme perturbations. FCIs for downregulation and upregulation of enzyme expression are plotted in blue and pink boxes, respectively. The mean of the FCI is presented as a dashed line and listed at the bottom (first row denotes upregulation FCI and second row denotes downregulation FCI).

To create better IPA producers, identification of potential targets for strain engineering is required. We therefore performed robustness analysis to identify potential modification targets on the pathway. We first estimated the system failure probability in response to the down- and upregulation of each pathway enzyme. This analysis is advisable because perturbations in gene expression levels may lead to disappearance of a steady state, for which we desire to foresee the outcome prior to in-depth metabolic engineering. Figure 3A shows the robustness profiles of the basis configurations for IPA production. The perturbation is defined as the fold change of enzyme expression over the reference state. The results suggest that in most cases, the pathway producing IPA has an increased probability of system failure if the enzyme level is too low (Fig. 3A). In those cases, metabolites for the reaction will be either depleted or overaccumulated by enzyme knockdown, which could form a so-called kinetic trap (13). On the upregulation side, the system robustness is very sensitive to the AcsA, which is a key subunit in acetyl-CoA synthase for CO_2_ reduction to CO. Our analysis suggested the biosystem may lose steady state upon AcsA upregulation, which could cause failure. We deduced that this sensitivity could corelate to the unique topology of the WLP. Note that the WLP consists of a methyl branch which contains cascade-like reactions connected in a series (Fig. 1A), while a carbonyl branch carries AcsA only. It is likely that the carbonyl branch lacks intermediate reactions that could buffer perturbation in kinetics changes. This indicates a potential risk of AcsA overexpression for strain engineering.

The ensemble modeling approach also yields the steady-state metabolic flux response to kinetic parameter perturbations. The simulation results of the ensemble can then guide the selection of potential targets for performance improvement. Based on the likelihood of a metabolic response in the ensemble, Fig. 3B shows the composite solution of multiple models of WLP and IPA synthesis after different degrees of enzyme activity perturbation. Only the models with a stable steady state are recorded. The intensity of color in Fig. 3B indicates the density of models or likelihood of results. For instance, in the case of AcsA, although overexpression of this gene increases the likelihood of system failure, the survival models in a middle range of upregulation (2-fold) still demonstrated much increased flux for IPA production (Fig. 3B). More quantitative results are presented in Fig. 3C, in which the FCIs of each pathway gene are statistically analyzed. AcsA’s FCI in upregulation is 0.12, 2- to 3-fold higher than that of most other genes (FCI, 0.04 to 0.06, Fig. 3C). However, note that the trade-off between performance and robustness will be a constraint to overexpress AcsA. As mentioned above, a higher AcsA activity results in much more likelihood of system failure (Fig. 3A). In comparison, AADC is also among the best ways to improve IPA flux (FCI_aadc_, 0.13; Fig. 3B and C), while its overexpression will not apparently increase the risk of system failure (Fig. 3A). Thus, it indicates that AADC could be a new engineering target, upregulation of which might enhance IPA productivity, but without robustness concerns.

### *In silico* analysis revealed a quantitative requirement of pathway enzymes.

We next carried out computational modeling for analyzing pathway enzymes’ minimum quantities that could afford unit IPA flux. Specifically, we sought the solution of the nonlinear optimization problem (18, 20) as described in equation 2 (see Materials and Methods). Kinetic parameters used in the enzyme-level expression are listed in Tables S2 and S3. It should be noted that this computational analysis takes both thermodynamic and enzyme kinetic constraints into account, thus providing a comprehensive description of fundamental enzyme requirements for maintaining a functional pathway, here, WLP for the conversion of CO_2_ to IPA (for modeling results see Table S4).

10.1128/msystems.01274-22.2 TABLE S2 Parameters for thermodynamics and enzyme protein cost analysis of the WLP-based IPA biosynthesis pathway in *C. ljungdahlii*. Standard Gibbs free energies (ΔG′^m^) were searched in the eQuilibrator database; Michaelis constants (*K_m_*), catalytic rate constants (*k*_cat_), and enzyme molecular weight (MW) were collected from the BRENDA database. If no data were available, default values of 200 s^−1^, 0.2 mM, and 40 kDa were assigned for *k*_cat_, *K_m_*, and MW, respectively. Note 1: The *k_m_* of AACT to acetate is quoted for *ctfAB* (1,200 mM) and *atoAD* (53.1 mM) as shown in the brackets. Download Table S2, PDF file, 0.2 MB. Copyright © 2023 Lo et al. 2023 Lo et al. https://creativecommons.org/licenses/by/4.0/ This content is distributed under the terms of the Creative Commons Attribution 4.0 International license.

10.1128/msystems.01274-22.3 TABLE S3 Parameters for thermodynamics and enzyme protein cost analysis of the Embden-Meyerhof-Parnas (EMP) pathway-based IPA biosynthesis pathway in *C. ljungdahlii*. Standard Gibbs free energies (ΔG′^m^) were searched in the eQuilibrator database; Michaelis constants (*K_m_*), catalytic rate constants (*k*_cat_) and enzyme molecular weight (MW) were collected from the BRENDA database. If no data were available, default values of 200 s^−1^, 0.2 mM, and 40 kDa were assigned for *k*_cat_, *K_m_*, and MW, respectively. Download Table S3, PDF file, 0.1 MB. Copyright © 2023 Lo et al. 2023 Lo et al. https://creativecommons.org/licenses/by/4.0/ This content is distributed under the terms of the Creative Commons Attribution 4.0 International license.

10.1128/msystems.01274-22.4 TABLE S4 Predicted enzyme protein cost for the conversion of CO_2_ to IPA through the Wood-Ljungdahl pathway. The enzyme amounts (g) are normalized to IPA flux (mol/s). Shown in columns 2 and 3 are the outcomes when *ctfAB* (C. acetobutylicum) and *atoAD* (E. coli source) are selected for aact, respectively. Other input of enzyme kinetics is as same as shown in Table S2. Download Table S4, PDF file, 0.09 MB. Copyright © 2023 Lo et al. 2023 Lo et al. https://creativecommons.org/licenses/by/4.0/ This content is distributed under the terms of the Creative Commons Attribution 4.0 International license.

**FIG 4:**
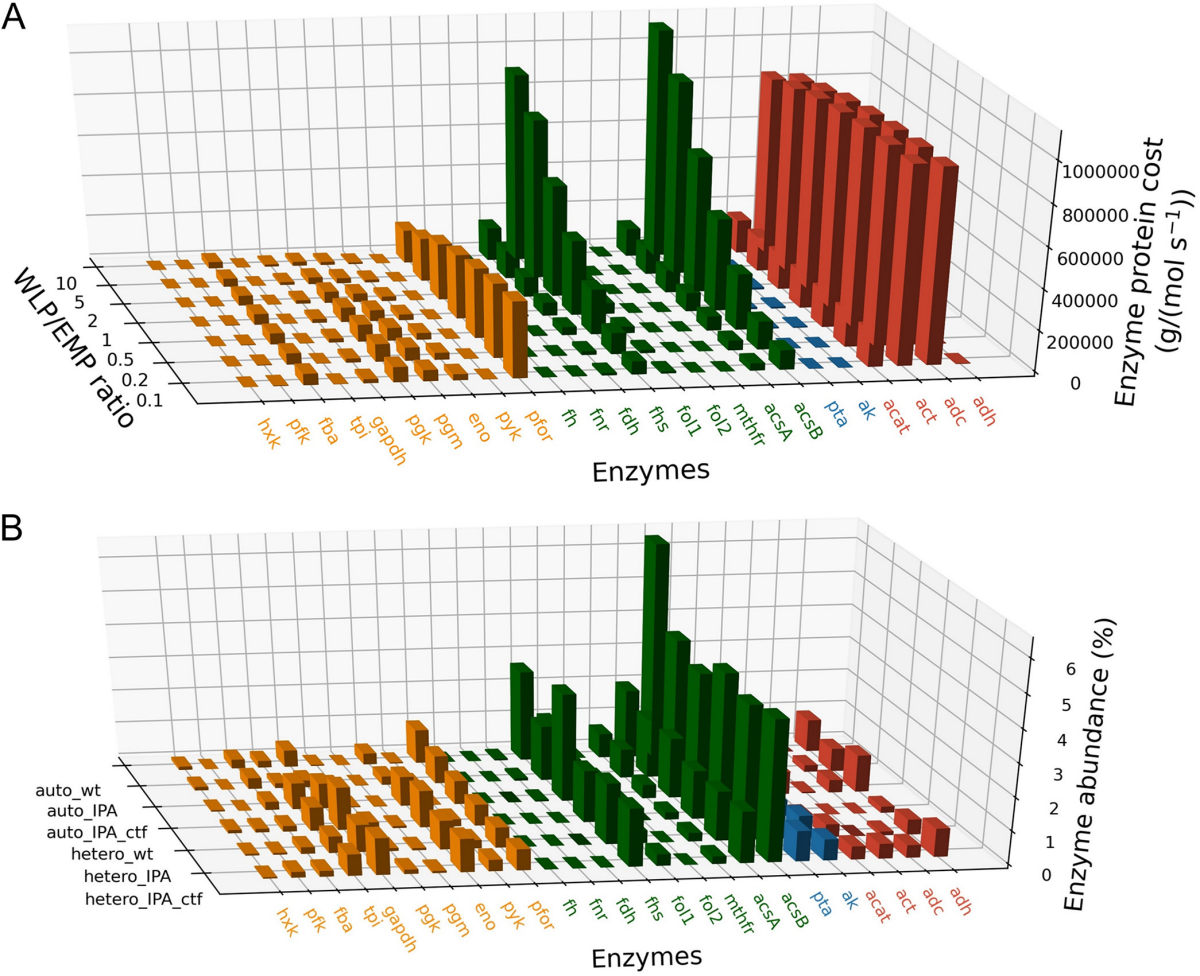
(A and B) Computational enzyme cost analysis (A) and experimental proteomics analysis (B) of the IPA synthesis pathway. (A) Enzyme protein costs denote the minimum enzyme mass required to support unit metabolic flux through the pathway and are estimated under various mixotrophic conditions represented by different ratios of pathway flux through the WLP and the Embden-Meyerhof-Parnas (EMP) pathway. Enzymes in the EMP pathway are labeled in orange, enzymes in the WLP are labeled in green, enzymes overexpressed for IPA synthesis in this study are labeled in red, and other enzymes are in blue. (B) Bar height denotes the abundance of enzymes in total protein measured by proteomic assay. IPA denotes the IPA v2 strain in which the IPA pathway was integrated into the genome. IPA_ctf denotes IPA v3-2, where the strain has an additional *ctfAB* gene introduced. Auto denotes an autotrophic condition in which cells were grown on the WLP by using syngas. Hetero denotes a heterotrophic condition in which cells were grown on fructose.

To consider a more realistic scenario, we must account for all important carbon fluxes generating IPA through acetyl-CoA. We analyzed the enzyme requirement from both the WLP and the glycolytic pathway toward IPA production, the relative flux between which would tailor the thermodynamic driving force and the metabolic outcomes. For example, higher WLP flux relative to glycolytic flux would hold a more negative thermodynamic potential and stronger CO_2_-fixing capabilities but produce less energetic currency ATP as a coproduct. The minimal enzyme protein requirement per unit IPA flux corelates with the ratio of WLP to glycolytic flux. Specifically, the larger amounts of enzymes will be needed when IPA is derived more from the WLP. This outcome aligns with the fact that rate-determining steps in the WLP would need higher enzyme quantities to compensate for the less efficient catalysis. Such a case was mirrored in AcsB and Fhs, where input *k*_cat_ 2.1 and 1.4, respectively, led to 2.0 × 10^6^ g/(mol s^−1^) of protein requirement, the top two among the WLP enzymes when the WLP dominates the flux for IPA synthesis (WLP/Embden-Meyerhof-Parnas [EMP] ratio equals 10). Interestingly, this protein cost analysis corroborated the proteome of *C. ljungdahlii*, in which AcsB and Fhs were also shown to be the most abundant proteins in the WLP (Fig. 4B).

The predictivity of the modeling outcome convinced us to focus on the engineered pathway more explicitly. As shown in Fig. 4A, under various mixotrophic conditions, heterologous enzymes in the synthetic IPA pathway require 2.9 × 10^6^ – 4.6 × 10^6^ g/(mol s^−1^) of protein, in which AACT accounts for 18.7 to 32.9% of total pathway protein cost, while AADC accounts for 18.4 to 32.4% of the pathway enzyme protein cost, followed by another heterologous enzyme (ACAT). The lower requirement of the ACAT enzyme than AACT and AADC is probably due to its better kinetic properties. We further compared the outcomes with the proteome data collected from the pathway host (Fig. 4B).

As shown in Fig. 4B, ACAT and AADC both were detected in the proteome of the engineered strain, while AACT was not measurable in proteomics. We inferred that AACT should be expressed due to its necessity in amount for IPA production. However, it was shown to have an inappreciable expression, which suggests a potential target for genetic engineering in the next engineering cycle.

### The next iteration of fine-tuning of the pathway led to a 2.8-fold increase in IPA production.

As analyzed above, we selected AADC and AACT as the targets for additional overexpression collectively. AADC was recommended by metabolic robustness analysis (Fig. 3A) as well as flux control analysis (Fig. 3B and 3C). AACT was selected because it was suggested as a key gene for IPA synthesis by protein cost analysis (Fig. 4A) but was not detected in the proteome of the first-generation IPA strain (Fig. 4B). For AACT overexpression, we considered a different gene option, *atoAD*, which was reported to encode a potentially better enzyme to catalyze the same reaction of AACT, as this gene product was shown to have a much lower *K_m_* (53.1 mM) for acetate than *ctfAB* (1,200 mM) and has been shown to yield higher IPA titer in other microbial chasses (19, 25, 26). Thus, we transformed constructs to determine whether overexpression of *atoAD* versus *ctfAB* was better at producing IPA (Fig. 2). The *aadc* was coengineered into both constructs.

The fermentation data showed that the construct with *ctfAB* and *aadc* produced 2.7 g/L IPA, while *atoAD* plus *aadc* produced 1.7 g/L IPA, both of which exceeded the highest titer of the first-generation engineered strain (<1 g/L) (Fig. 2). These results implied that (i) *ctfAB* and *aadc* overexpression are indeed able to increase IPA flux, as higher amounts of IPA were produced than in the parental strain, consistent with the prediction by systems biology and modeling analysis, and (ii) the *atoAD* overexpression is inferior to the *ctfAB* overexpression. Despite a lower *K_m_* of *atoAD* for acetate, which may lead to a lower enzyme amount for catalysis, the acetogenic host can maintain acetate at a high level, thus satisfying optimal working conditions for *ctfAB*. A much higher acetate concentration presumably resulted in competitive substrate inhibition as reported for *atoAD* (26).

To verify the dosage increase of pathway enzymes, another set of proteomics data was generated on the baseline IPA strain with the overexpression of CtfAB and AADC (Fig. 4B, IPA_ctf). In comparison to the IPA baseline strain where CtfAB was detected, we identified much improved protein abundance of CtfAB, showing the direct outcome of our genetic engineering efforts. This result aligns with improved IPA titer (2.7 g/L). Overall, based on these engineering efforts, the new IPA strain has successfully achieved a 2.8-fold increase in IPA production. This progress validates that strain development of *C. ljungdahlii* can be directed by thermodynamic and kinetic models.

### The rationally engineered strain has good potential for IPA production under various gas-fermenting conditions.

**FIG 5:**
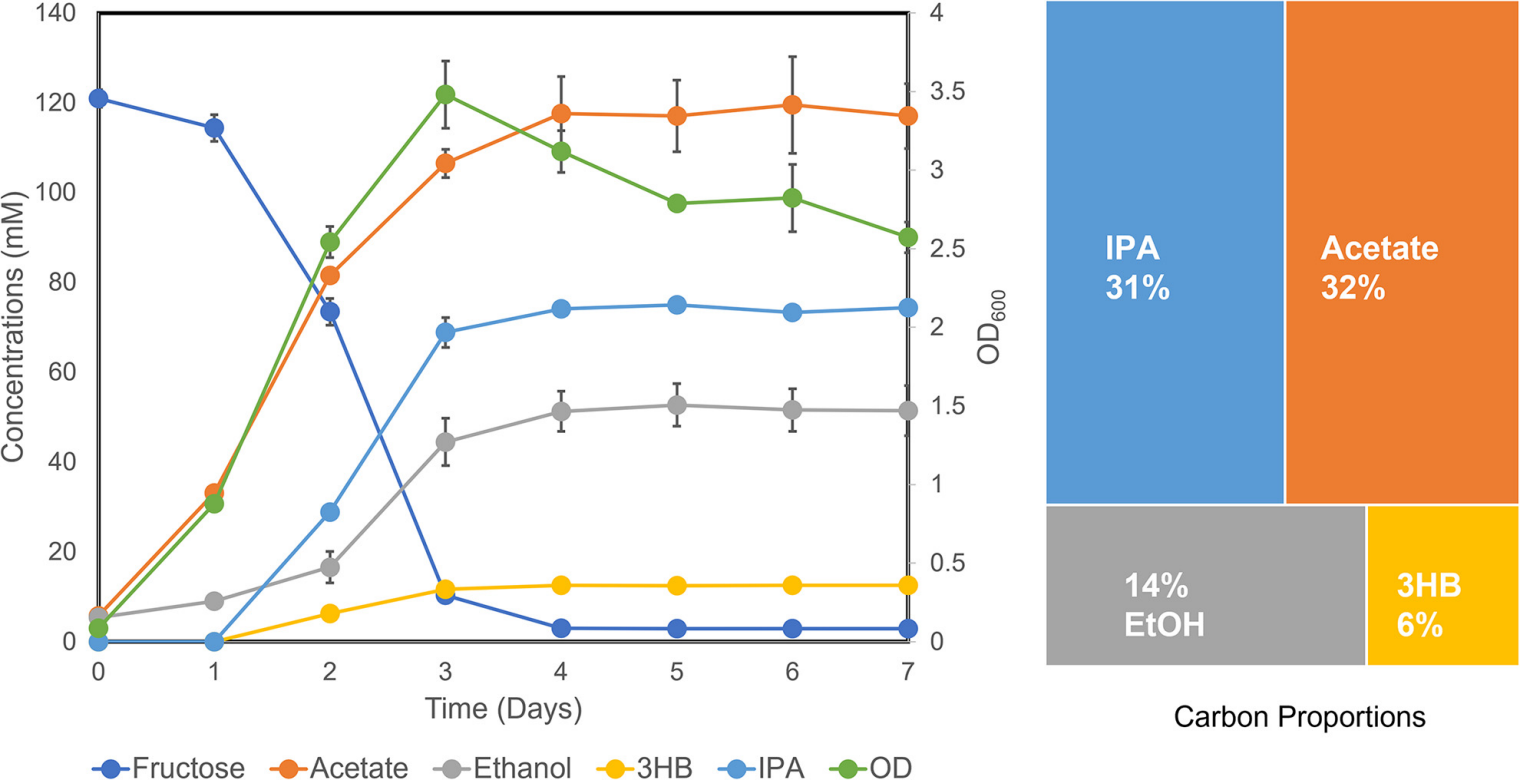
Fermentation kinetics of the IPA v3-2 strain grown under the mixotrophic condition with 20 g/L fructose fed in the medium as well as 6 lb/in2 syngas (CO/CO_2_/H_2_) in the headspace.

Next, we found that the rationally engineered strain (IPAv3-2) is metabolically flexible in various environments and may have exceptional IPA production potentials, for example, under the mixotrophic and autotrophic growth condition. Mixotrophic growth by acetogens allows for improved carbon conversion due to their ability to coutilize both sugars and gaseous carbon simultaneously (7). We investigated if improved IPA titers can be achieved by growing *C. ljungdahlii* IPA v3-2 strain with 20 g/L fructose supplemented with a syngas mixture containing 50%/30%/20% CO/CO_2_/H_2_ in the headspace. Cells were grown for 7 days, and products were analyzed. Mixotrophic growth produced a good number of cells, with the highest optical density of 3.48 achieved at day 3 (Fig. 5). Also, mixotrophic growth showed an improvement of final IPA titers, which were 74 ± 1.3 mM (4.44 ± 0.078 g/L). Acetate was the major product, which reached 117 mM, after 7 days. Both ethanol and 3-hydroxybutyrate (3HB) were produced by the culture, which reached titers at around 50 mM and 12 mM, respectively. Overall, mixotrophic growth showed a high carbon yield for all products (acetate, ethanol, IPA, and 3HB), accounting for 83% carbons from fructose. This number exceeds the theoretical maximum of the glycolytic EMP pathway (66.7%), aligning with the fact that CO_2_ released from the EMP pathway was effectively recycled by the carbon-fixing WLP. Compared to all other fermentation products, the proportion of IPA reached 31% of the total by the end of the experiment, indicating that pathway engineering has led IPA to become the equivalently largest carbon sink in this biocatalytic process, comparable to the natural acetogen’s product (acetate, 32%).

**FIG 6:**
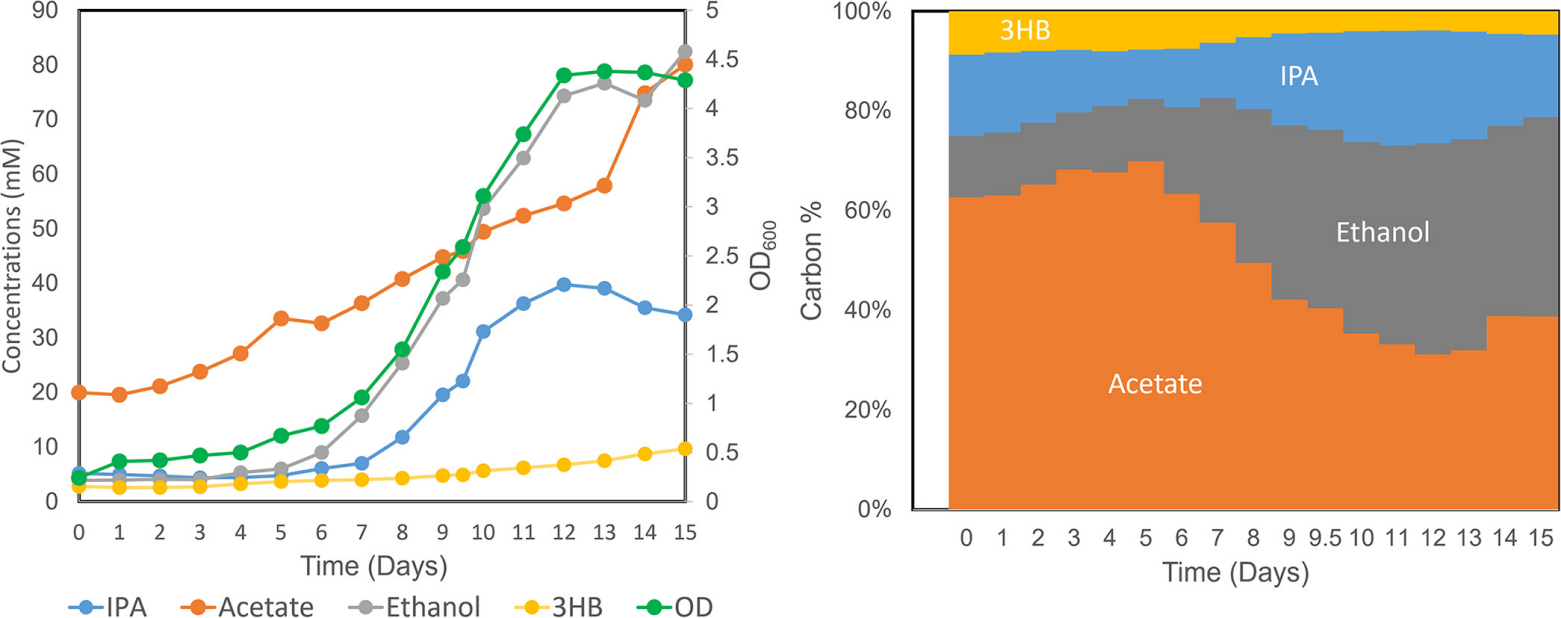
Time course of the IPA v3-2 strain grown autotrophically in a 2-L gas-fermenting bioreactor sparged with syngas (CO/CO_2_/H_2_). Shown on the left are the cell OD_600_ and production of IPA, acetate, ethanol, and 3-hydroxybutarate over time. Shown on the right are the changes of carbon molar yield in the products.

To further investigate the rationally engineered strain in a syngas-fermenting environment, we inoculated the IPA v3-2 into YT medium (containing Yeast extract and Tryptone but without fructose feeding) and performed a fermentation experiment in a 2-L bioreactor. This gas fermentation system has recently achieved a high-titer production of nonnative 3-hydroxybutyrate from *C. ljungdahlii* (27). Sparged with a constant syngas mix (CO/CO_2_/H_2_: 70%/20%/10%), the fermentation results were recorded daily and are shown in Fig. 6. Host cells underwent an initial ~100-h lag growth phase and then grew exponentially, reaching the highest optical density (OD) of around 4 to 5 at day 13. Associated with cell growth, the IPA production reached the maximum at day 12, demonstrating a peak titer (Titer_max_) of 39.7 mM (2.39 g/L). In terms of carbon partitioning, IPA accounted for up to 29% of carbon molarity of all products. This value was defined as the maximum carbon proportion during the fermentation (C-Proportion_max_). In addition, acetate production continued over time and reached its highest level (80.1 mM; C-Proportion_max_, 34%) at the end of fermentation. This result is in line with the fact that acetogenic bacteria require steady acetate production to compensate for the bioenergetics limit. Interestingly, we also observed a significant amount of ethanol being produced in the process (Titer_max_, 82 mM; C-Proportion_max_, 35%). This phenotype was not typically consistent with a native *C. ljungdahlii* strain in which ethanol was only moderately generated as a secondary product. Our results imply that the redox status might have been changed substantially in the engineered strain. Ethanol production may have been selected to balance electron flow and carbon metabolism. Conclusively speaking, although strain development will be further pursued, our rational design has provided a solid platform for converting syngas to value-added alcohols.

## DISCUSSION

Gas fermentation for the production of nonnative chemicals has received significant attention in recent years. This has led to remarkable progress in strain development (9, 12), but improvements are still needed to make the process industrially viable. As acetogens are difficult to rapidly engineer and test on syngas, tools are needed to generate informed approaches for strain engineering. Recently, Liew et al. described methods to overcome technical hurdles in Clostridium autoethanogenum, to achieve carbon-negative production of acetone and IPA at industrially relevant selectivity and scale (12). The pronounced approaches they used for strain engineering include combinatorial library construction and iPROBE (*in vitro* prototyping and rapid optimization of biosynthetic enzymes), which were particularly useful in screening candidate enzymes for pathway optimization. In comparison, we adopted a model-driven strain redesign approach for rational engineering, which allowed for significant improvements in our iterative IPA-producing strains. In this study, we used thermodynamics analysis, enzyme protein cost analysis, and proteomics to inform strain redesigns and optimizations for increasing IPA production in the gas-fermenting organism *C. ljungdahlii*. A key finding in this contribution is that we predicted that AACT and AADC are the two key IPA rate-limiting reactions along the central metabolism and the IPA pathway. Overexpression of these enzymes indeed leads to an increase in the downstream IPA flux. These studies indicate that our developed computational workflow for the integration of thermodynamics, enzyme kinetics, and ensemble modeling for *C. ljungdahlii* is a valuable method to pinpoint key enzyme expressions for the optimization of the IPA flux. Currently, this work solely considered IPA and the primary pathways in the central metabolism of the acetogen (e.g., WLP and the EMP pathway). Given a simplified central metabolic pathway, we can obtain reasonable flux control coefficients (FCCs) that actively increase the flux of the product IPA. Probing how they all interact at the genome-scale level will provide more information for realistic future strain designs that are physically capable of growing under real conditions. The insights gained from this relatively smaller model provide us with the ability and understanding to expand further onto a genome-scale metabolic model for a much broader analysis of how FCCs could affect the production of IPA in the context of the entire genome.

Also, as a next step, we are interested in developing more effective genome engineering tools that can precisely and quantitatively modify the target pathway enzyme levels. Comparing the optimization goal provided by computational modeling and realistic enzyme expression levels in the -omics data sets (e.g., as shown in Fig. 4), there is still adequate space for the pathway enzymes to be iteratively improved. Better engineering strategies and tools for gas-fermenting microbial chasses will accelerate the process toward this goal. The tools developed in this study are applicable to a diverse array of microbes yielding various targeted products, hence affording a broader impact in the field of biofuels and bioenergy production.

## MATERIALS AND METHODS

### *In silico* analysis.

**Pathway thermodynamics and enzyme protein cost analysis.** Thermodynamics and enzyme protein cost analysis were applied to assess the feasibility and protein requirement of the designed metabolic pathway. Pathway feasibility was evaluated by solving a max-min driving force (MDF) problem which seeks to maximize the Gibbs energy change, ΔG′, of the most thermodynamically unfavorable reaction by tuning the concentrations of intermediates. The problem is defined as (18):

(1) $$\begin{array}{l} \underset{\ln(c)}{\max}\;\min(-\Delta G_{1}^{\prime },-\Delta G_{2}^{\prime },\ldots ,-\Delta G_{m}^{\prime })\\ \;s.t.\;\Delta G^{\prime }=\Delta G^{\prime 0}+R\cdot T\cdot S\cdot \ln(c)\;\\ \;\ln(c_{\min})\leq \ln(c)\leq \ln(c_{\max}) \end{array}$$

where *m* is the number of pathway enzymes, **c** is the vector of involved metabolite concentrations with upper bounds and lower bounds as **c**_min_ and **c**_max_, respectively, and **S** is the stoichiometric matrix of the pathway reactions.

In contrast, enzyme protein cost analysis aims to estimate the minimal protein mass required to support a unit pathway flux by solving a nonlinear optimization problem (18, 20):

(2) $$\begin{array}{l} \underset{\ln(c)}{\min}\;\Lambda =\frac{\sum_{i}^{m}\left(MW_{i}\cdot E_{i}\right)}{v_{\mathrm{in}}}\\ \;s.t.\;\ln(c_{\min})\leq \ln(c)\leq \ln(c_{\max}) \end{array}$$

where Λ is the enzyme protein requirement, *v*_in_ denotes influx to a pathway, MW is the enzyme molecular weight, and E is the expression of the reaction enzyme level derived from a common modular rate law (28) and Haldane relationship (29).

The calculation was performed using our previously developed pathway analysis tool, PathParser (18). The thermodynamic and kinetic parameters of enzymes in the WLP and EMP-based IPA biosynthesis pathway are listed in Tables S2 and S3. The standard Gibbs free energies (ΔG′^m^) were searched in the eQuilibrator database (30). ΔG′^m^ of the fructose phosphotransferase (PTS) system was estimated using equilibrator-api (31). Michaelis constants (*K_m_*), catalytic rate constants (*k*_cat_), and enzyme molecular weights (MW) of *C. ljungdahlii*, Moorella thermoacetica, Acetobacterium woodii, Clostridium formicoaceticum (32), and E. coli were preferentially chosen from BRENDA (33). If no data are available, default values of 200 s^−1^, 0.2 mM, and 40 kDa were used, respectively. These numbers were assigned for metabolic modeling (8, 20). Their validity is based on statistical analysis of a large number of known metabolic enzymes in the BRENDA database. In MDF optimization of the WLP-based IPA synthesis pathway, concentrations of involved metabolites were constrained to vary between 1 μM and 10 mM. For enzyme protein cost estimations under various mixotrophic conditions, a series of split ratios 10, 5, 2, 1, 0.5, 0.2, and 0.1 were assigned to the WLP- and EMP-producing unit amount of IPA. Upper bound metabolite concentration constraints were relaxed to 100 mM for appropriate solutions with physiological significance. The above-described linear programming and nonlinear programming problems were both solved using an open-source Python optimization package, openopt.

### Metabolic robustness analysis.

Pathway robustness and flux changes against enzyme perturbations were evaluated using an ensemble modeling approach combined with the continuation method (13). First, an ensemble of models was generated with log-uniformly sampled kinetic parameters from the feasible spaces, which meanwhile is subjected to the same flux distribution of the reference state (34, 35). Then the continuation method was used to simulate the system response to enzyme expression perturbations. The dynamic system of metabolite concentrations can be expressed as:

(3) $$\frac{dc}{\mathrm{dt}}=f(c,E)=S^{T}\cdot v(c,E)$$

where **f** denotes the derivative of the concentrations with respect to time, which is a function of metabolite concentrations **C** and enzyme levels **E**. At the metabolic steady state, **C** is constant, and the derivative of **C** with respect to *t* equals zero. Accordingly, the derivative of **f**(**c**, **E**) with respect to **E** equals zero too, which yields (13, 18):

(4) $$\frac{dc_{\mathrm{SS}}}{dE}=-{\left(\frac{\partial f}{\partial c_{\mathrm{SS}}}\right)}^{-1}\cdot \frac{\partial f}{\partial E}$$

where **c**_SS_ denotes metabolite concentrations at the steady state. The Jacobian matrix $\frac{\partial f}{\partial c_{\mathrm{SS}}}$ determines the metabolic robustness of a pathway in which a system failure occurs if the real part of any of the Jacobian eigenvalues pass through zero.

The above-described differential equations are solved along with up- and downregulation of enzyme levels until system failure happens, and the number of models is counted to calculate the probability of system failure for each enzyme at various enzyme levels. When solving the equations, metabolite concentrations will be updated, and pathway fluxes can be reestimated accordingly, responding to enzyme perturbations. Robustness and flux response analysis of native WLP and the IPA pathway were also performed using PathParser (18) with an ensemble of 100 generated models.

### Experimental section.

**Strains, media, and chemicals.** *C. ljungdahlii* DSM 13528 was purchased from the Leibniz Institute DSMZ (Braunschweig, Germany). The YTF rich medium, consisting of 10 g L^−1^ Bacto yeast extract, 16 g L^−1^ Bacto tryptone, 4 g L^−1^ NaCl, 5 g L^−1^ fructose, and 0.5 g L^−1^ cysteine-HCl, and the defined PETC medium (ATCC 1754, American Type Culture Collection, Manassas, VA) were utilized for growing *C. ljungdahlii*. Cell growth was monitored at 600 nm with a DU 800 spectrophotometer (Beckman-Coulter, Brea, CA). All chemical reagents used in the growth studies were purchased from Sigma-Aldrich, except Bacto yeast extract and tryptone, which were purchased from Becton, Dickinson.

### Construction of IPA producer strains in *C. ljungdahlii*.

Standard molecular techniques with enzymes and Escherichia coli NEB 10-beta were from New England Biolabs (NEB). Plasmid pMTL80000 series modular plasmids were from Chain Biotech (Nottingham, UK) and were used to generate the constructs transformed into *C. ljungdahlii*. Plasmids were generated using previously established protocols (36). Briefly, amplicons were generated by PCR, which were then ligated together using Gibson Assembly (NEB). Table 1 lists the primers used for cloning. The plasmid sequence was confirmed by sequencing and transformed into the wild-type *C. ljungdahlii*.

**TABLE 1:** Primers used for the PCR amplification and cloning

Primer	Sequence (5′–3′)	Note
XJL.683.f	TTCGTCTTCACCTCGAGCCTGCAGGGGCATTTTCAAAGAAATAACTAG	Pfdx primers
XJL.684.r	TACACAATTTTTcatCTTATGTAACACCTCCTTAATTTTTAG	
XJL.685.f	GTGTTACATAAGatgAAAAATTGTGTAATAGTATCAGC	*atoB* primers
XJL.690.r	CTTACCTCCTCCTCCTAATTGGATAttaATTTAATCTTTCTATTACCATAG	
XJL.691.f	TATCCAATTAGGAGGAGGAGGTAAGATGAACTCTAAAATAATTAGATTTG	*ctfAB* primers
XJL.692.r	AGCCCTACCTCCTTTATTCGGTGCTCTAAACAGCCATGGGTCTAAGTTC	
XJL.693.f	AGCACCGAATAAAGGAGGTAGGGCTATGTTAAAGGATGAAGTAATTAAAC	aadc primers
XJL.694.r	ACGAGTCCTTTAGACTTTACTATCTTTACTTAAGATAATCATATATAACTTCAGC	
XJL.738.f	AGATAGTAAAGTCTAAAGGACTCGTATGAAAGGTTTTGCAATGTTAG	sadh primers
XJL.739.r	AGGATCCCCGGGTACCGAGTTAGAATGTAACTACTGATTTAATTAAATCTTTTG	
XJL.798.f	ATGACCATGATTACGAATTCGAGATGGATAATTTAGTTATAAATACATTG	*pyrE* 300-bp primers
XJL.746.r	CTCGAATTCGTAATCATGGTCATTTATCCCCTTCTTATAGTCATATTTC	
XJL.747.f	CTCGGTACCCGGGGATCCTCTAGGTCGAAAAAATCAATGCACGATGCAG	*pyrE* 1,200-bp primers
XJL.747a.r	CTAGAGGATCCCCGGGTACCGAGGGAACCTGATGCCGTTAAATAA	
XJL.675.f	accatgattacgaattcgagTTAATATGCCGACCACGTTG	Clo1313_1194 primers
XJL.762.r	CTAATTATTTTAGAGTTCATAGTTTTTTTCCCCCTTTAATG	
XJL.763.f	GACCCATGGCTGTTTAGAGGGCATTTGAAAAAATAGG	Ppta primers
XJL.757.r	cctttaacatGTTCATTTCCTCCCTTTAAATTTAAC	
XJL.758.f	ggaaatgaacATGTTAAAGGATGAAGTAATTAAAC	aadc primers for V2
XJL.759.r	xc	
XJL.760.f	gaaaaaaactATGAACTCTAAAATAATTAGATTTGAAAATTTAAG	*ctfAB* primers for V2
XJL.761.r	caaatgccctCTAAACAGCCATGGGTCTAAG	
XJL.754.f	gaaaaaaactATGAAAACTAAATTGATGACTTTAC	*atoAD* primers for V2
XJL.755.r	caaatgccctTCATAGATCACCCCTCTG	
*pyrE*.KO.F	AGAGGAATAATTTAGGAGGACAG	*pyrE* 300-screen primers
seq Ec *atoB* R	CAGATTCTATTGCTGCTGCTGC	
XJL.748.r	CCAGTAGAAGGATGCACC	*pyrE* 1200-screen primers

Transformation was based on previously reported protocols (37). Briefly, cultures were grown in 40 mM DL-threonine to the mid-log phase (OD, 0.4 to 0.8) and then harvested by centrifugation. Cells were washed twice with ice-cold SMP buffer (270 mM sucrose, 1 mM MgCl_2_, 7 mM sodium phosphate, pH 6) and then resuspended in SMP buffer with 10% dimethyl sulfoxide (DMSO) and stored at −80°C until being used for transformation. Electroporation was performed in a Coy anaerobic chamber with a Gene Pulser Xcell Bio-Rad electroporator (Hercules, CA) with the following settings: in a 1-mm cuvette, 25 μL of cells were mixed with 2 to 5 μg of DNA, pulsed at 625 kV with resistance at 600 Ω and a capacitance of 25 μF. Cells were then resuspended in 5 mL of YTF and recovered overnight at 37°C. Cells were plated in 1.5% agar YTF with thiamphenicol (Tm) at a concentration of 10 μg/mL. Integration of the IPA pathway into the *pyrE* locus was based on previously established methods (38). Briefly, cells were subcultured in YTF medium with Tm and then plated on YTF agar with Tm and 5-fluoroorotic acid (5FOA) at a final concentration of 500 μg/mL. 5FOA-resistant single colonies were screened for genome integration using PCR and then subcultured in YTF until Tm sensitive colonies were isolated.

### Mixotrophic growth of engineered strains.

Our rationally engineered strain was initially grown in 50 mL YTF plus Tm seed cultures overnight. After overnight growth, cells were inoculated into 50 mL YTF plus Tm plus 1 g/L CaCO_3_ (for rough pH maintenance) using a 250-mL Duran pressure plus bottle (DWK Life Sciences, USA) as the culture vessel. A syngas mixture containing 50%/30%/20% CO/CO_2_/H_2_ was added at 6 lb/in^2^ to mixotrophic bottles. Optical density and high-performance liquid chromatography (HPLC) samples were taken daily. The syngas mixture was refilled as necessary every day to maintain 6 lb/in^2^ for the mixotrophic conditions.

### Gas fermentation in the bioreactor.

Seed cultures were prepared for the bioreactor by adding 10 mL overnight growth of the IPA v3.2 strain to 50 mL YT medium containing thiamphenicol in a 250-mL pressure plus bottle and pressurized to 8 lb/in^2^ with a syngas mix of 70%/20%/10% CO/CO_2_/H_2_. Seed cultures were left to grow at 37°C with shaking at 250 rpm until all the syngas was consumed. IPA production was verified during this growth by high-performance liquid chromatography (HPLC). Once sufficient syngas had been consumed, the seed cultures were transferred into the 2-L bioreactor. The 2-L bioreactor contained 1.7 L YT medium with thiamphenicol and was sparged with a constant syngas mix (70%/20%/10% CO/CO_2_/H_2_) at a rate of 300 sccm (standard cubic centimeter per minute). The pH was controlled to 5.2 with an initial stirring speed of 300 rpm. This was increased gradually to 900 rpm as the OD increased during growth. The fermentation results were recorded daily by OD at 600 nm (OD_600_) measurement and HPLC analysis.

### Analysis of fermentation products.

Fermentation liquid samples of 150 μL were extracted by syringe, filtered using Costar Spin-X 0.45-μm filters (Corning, Corning, NY), and stored at −20°C until the experiments were completed. Fermentation products in the liquid phase (acetate, ethanol, IPA, and acetone) were measured by HPLC on a 1,200 series Agilent device (Santa Clara, CA) with an Aminex HPX-87H column using a Micro Guard Cation H cartridge at 55°C with 4 mM H_2_SO_4_ mobile phase.

### Proteome measurement and analysis.

The proteomic analysis of wild-type and IPA strains was conducted following the same method as in our previous report (18). First, 10 μg of trypsin-digested peptides from each sample was loaded onto a C_18_ capillary column coupled to a Thermo LTQ Orbitrap mass spectrometer (Thermo Scientific, Rockford, IL). The peptide identity was analyzed at a resolution of 30,000. Dynamic exclusion was enabled in this case with the setup of a repeat count of 1, repeat duration of 30 s, and exclusion duration of 90 s. The peptide identity was obtained by searching the tandem MS spectra using PatternLab for proteomics (39). Protein abundances were quantified based on spectral count. Each MS spectrum represents a peptide, which can be identified by matching the experimental MS spectrum with the theoretical spectrum based on constraints set in the data searching algorithm. For each protein, its abundance is represented by spectral counts from all of its identified peptides. To normalize the protein size differences and protein number differences among replicates, the normalized spectral abundance factor (NSAF) (40) was used to compare between the control group and the experimental group. The mass spectrometry data for proteomics have been deposited to the MassIVE repository with the data set identifier MSV000088839 and the ProteomeXchange Consortium (41) with the data set identifier PXD031695.
