# Supplementary material for: Thermodynamic and Kinetic Modeling Directs Pathway Optimization for Isopropanol Production in a Gas-Fermenting Bacterium
Source: mSystems. 2023 Mar 27;8(2):e01274-22. doi: 10.1128/msystems.01274-22 (PMC10134883; doi:10.1128/msystems.01274-22)
Supplement: TABLE S1 [file msystems.01274-22-s0001.pdf]

**Table S1.**

| Metabolite | Optimized Concentration (mM) |
|---|---|
| Ac | 10.000 |
| AcAc | 0.001 |
| AcAcCoA | 0.019 |
| AcCoA | 10.000 |
| Ace | 0.642 |
| AcP | 0.206 |
| ADP | 0.985 |
| ATP | 0.010 |
| CO | 0.056 |
| CO2 | 5.027 |
| CoA | 0.001 |
| Fm | 1.601 |
| FormylTHF | 0.114 |
| IPA | 0.012 |
| MethenylTHF | 0.002 |
| MethyleneTHF | 0.002 |
| MethylTHF | 5.943 |
| NAD | 0.002 |
| NADH | 4.989 |
| NADP | 0.136 |
| NADPH | 0.074 |
| oFdx | 0.004 |
| Pi | 0.641 |
| rFdx | 2.387 |
| THF | 5.068 |
