# Supplementary material for: Thermodynamic and Kinetic Modeling Directs Pathway Optimization for Isopropanol Production in a Gas-Fermenting Bacterium
Source: mSystems. 2023 Mar 27;8(2):e01274-22. doi: 10.1128/msystems.01274-22 (PMC10134883; doi:10.1128/msystems.01274-22)
Supplement: TABLE S2 [file msystems.01274-22-s0002.pdf]

Table S2.

Enzyme ID	ΔG'm (kJ mol^{-1})	Substrates	Products	Substrate K_m (mM)	Product K_m (mM)	k_{cat} (s^{-1})	Enzyme MW (kDa)
fdh	14.8	CO2,NADH	Fm,NAD	0.2;0.05	0.2;0.2	6.2 [1]	80.7
fhs	-7.2	THF,Fm,ATP	FormylTHF,ADP,Pi	0.22;8.2;0.1555	10;0.06;5	1.4 [1]	240
fol1	3	FormylTHF	MethenylTHF	0.2	0.19	200	41
fol2	-11.7	MethenylTHF,NADPH	MethyleneTHF,NADP	0.057 [4];0.029 [4]	0.127;0.0595	1600 [2]	70
mthfr	-43.2	MethyleneTHF,NADH	MethylTHF,NAD	0.001 [4];0.026 [4]	0.12;0.2	324 [3]	124
acsA	28.4	CO2,rFdx,0.5NADH	CO,oFdx,0.5NAD	0.2;0.2;0.2	0.2;0.2;0.2	10 [5]	67.955
acsB	-37.9	MethylTHF,CoA,CO	THF,AcCoA	0.2;0.2;0.2	0.2;0.2	2.1 [6]	83.554
pta	11.3	AcCoA,Pi	CoA,AcP	0.02 [4];2.1 [4]	0.56;0.66	135.2 [4]	38
ak	-12.6	ADP,AcP	ATP,Ac	3.355;0.58	1.435;116.5	1261.7	88
acat	24.9	2AcCoA	CoA,AcAcCoA	0.2552 [4]	0.008 [4];0.0176 [4]	6.5 [4]	161.92
aact	-6.1	AcAcCoA,Ac	AcAc,AcCoA	0.2;1200 [7] (53.1 [4])	0.2;0.2	200	48.006
aadc	-38.1	AcAc	CO2,Ace	6.3	0.2;0.2	507.3	355.12
sadh	-8	Ace,NADPH	IPA,NADP	0.6;0.2	0.2;0.2	200	95

[1] Data from *Moorella thermoacetica* (1).
[2] Data from *Acetobacterium woodii* (1).
[3] Data from *Clostridium formicoaceticum* and *Moorella thermoacetica* (1).
[4] Data from *Escherichia coli* (Brenda database).
[5] Data from (2).
[6] Data estimated from (3).
[7] Data from *Clostridium acetobutylicum* (Brenda database).

References:

1. Ragsdale SW, Pierce E. 2008. Acetogenesis and the Wood–Ljungdahl pathway of CO2 fixation. Biochimica et Biophysica Acta (BBA) - Proteins and Proteomics 1784:1873-1898.
2. Can M, Armstrong FA, Ragsdale SW. 2014. Structure, Function, and Mechanism of the Nickel Metalloenzymes, CO Dehydrogenase, and Acetyl-CoA Synthase. Chemical Reviews 114:4149-4174.
3. Roberts JR, Lu WP, Ragsdale SW. 1992. Acetyl-coenzyme A synthesis from methyltetrahydrofolate, CO, and coenzyme A by enzymes purified from Clostridium thermoaceticum: attainment of in vivo rates and identification of rate-limiting steps. Journal of Bacteriology 174:4667-4676.
