# Supplementary material for: Thermodynamic and Kinetic Modeling Directs Pathway Optimization for Isopropanol Production in a Gas-Fermenting Bacterium
Source: mSystems. 2023 Mar 27;8(2):e01274-22. doi: 10.1128/msystems.01274-22 (PMC10134883; doi:10.1128/msystems.01274-22)
Supplement: TABLE S3 [file msystems.01274-22-s0003.pdf]

Table S3.

Enzyme ID	$\Delta G'^{m}$ (kJ mol^{-1})	Substrates	Products	Substrate K_m (mM)	Product K_m (mM)	k_{cat} (s^{-1})	Enzyme MW (kDa)
pts	-41.7	Fructose,PEP	F1P,Pyr	0.2;0.2	0.2;0.2	200	57.5
pfk	-10.8	F1P,ATP	FBP,ADP	0.62;0.14	0.2;0.2	200	62.9
fba	3.4	FBP	GAP,DHAP	0.17 [1]	0.2;0.2	10.8 [1]	33.579
tpi	5.2	DHAP	GAP	0.2	1.03 [1]	9000 [1]	27.172
gapdh	32.3	GAP,Pi,NAD	BGP,NADH	0.89 [1];0.2;0.53 [1]	0.2;0.2	200	38.575
pgk	-17.8	BGP,ADP	G3P,ATP	0.2;0.2	0.2;0.2	200	42.956
pgm	5	G3P	G2P	4.7	0.2	200	56.3
eno	-3.8	G2P	PEP	0.2	0.2	200	46.94
pyk	-31.2	PEP,ADP	Pyr,ATP	0.2;0.2	0.2;0.2	200	63.207
pfor	-13.2	Pyr,CoA,2oFdx	CO2,AcCoA,2rFdx	0.2;0.2;0.2	0.2;0.2;0.2	200	129.94
pta	11.3	AcCoA,Pi	CoA,AcP	0.02 [1];2.1 [1]	0.56;0.66	135.2 [1]	38
ak	-12.6	ADP,AcP	ATP,Ac	3.355;0.58	1.435;116.5	200	88
acat	24.9	2AcCoA	CoA,AcAcCoA	0.2552 [1]	0.008 [1];0.0176 [1]	6.5 [1]	161.92
aact	-6.1	AcAcCoA,Ac	AcAc,AcCoA	0.2;1200 [2]	0.2;0.2	200	48.006
aadc	-38.1	AcAc	CO2,Ace	6.3	0.2;0.2	507.3	355.12
sadh	-8	Ace,NADPH	IPA,NADP	0.6;0.2	0.2;0.2	200	95

[1] Data from *Escherichia coli* (Brenda database).
[2] Data from *Clostridium acetobutylicum* (Brenda database).
