# Supplementary material for: Thermodynamic and Kinetic Modeling Directs Pathway Optimization for Isopropanol Production in a Gas-Fermenting Bacterium
Source: mSystems. 2023 Mar 27;8(2):e01274-22. doi: 10.1128/msystems.01274-22 (PMC10134883; doi:10.1128/msystems.01274-22)
Supplement: TABLE S4 [file msystems.01274-22-s0004.pdf]

Table S4.

#Reaction	Optimized Enzyme Cost (g/mol/s)	
fdh	26086.99	26095.99
fhs	342910.87	342920.52
fol1	459.74	470.50
fol2	88.81	88.96
mthfr	765.45	765.45
acsA	13591.05	13591.05
acsB	88929.28	83650.47
pta	324.41	331.86
ak	489.07	497.78
acat	32149.92	28438.45
aact*	9545.82	4233.70
aadc	9985.67	4715.71
sadh	475.01	475.01
Total	525802.09	506275.44
